# Sex Differences in Clinical Characteristics, Management Strategies, and Outcomes of STEMI With COVID-19: NACMI Registry

**DOI:** 10.1016/j.jscai.2022.100360

**Published:** 2022-05-19

**Authors:** Odayme Quesada, Logan Van Hon, Mehmet Yildiz, Mina Madan, Cristina Sanina, Laura Davidson, Wah Wah Htun, Jacqueline Saw, Santiago Garcia, Payam Dehghani, Larissa Stanberry, Anna Bortnick, Timothy D. Henry, Cindy L. Grines, Catherine Benziger

**Affiliations:** a Women’s Heart Center, The Christ Hospital Heart and Vascular Institute, Cincinnati, Ohio; b The Carl and Edyth Lindner Center for Research and Education, The Christ Hospital, Cincinnati, Ohio; c Essentia Health, Duluth, Minnesota; d Schulich Heart Centre, Sunnybrook Health Sciences Centre, University of Toronto, Toronto, Ontario, Canada; e Montefiore Medical Center, Bronx, New York; f Northwestern University Feinberg School of Medicine, Chicago, Illinois; g Gundersen Health System, La Crosse, Wisconsin; h Vancouver General Hospital, Vancouver, British Columbia, Canada; i Prairie Vascular Research Inc, Regina, Saskatchewan, Canada; j Minneapolis Heart Institute Foundation, Minneapolis, Minnesota; k Northside Cardiovascular Institute, Atlanta, Georgia

**Keywords:** COVID-19, mortality, sex differences, STEMI

## Abstract

**Background:**

Women with ST-segment elevation myocardial infarction (STEMI) had worse outcomes than men prior to the COVID-19 pandemic. Although concomitant COVID-19 infection increases mortality risk in STEMI patients, no studies have evaluated sex differences in this context.

**Methods:**

The North American COVID-19 STEMI registry is a prospective, multicenter registry of hospitalized STEMI patients with COVID-19 infection. We compared sex differences in clinical characteristics, presentation, management strategies, and in-hospital mortality.

**Results:**

Among 585 patients with STEMI and COVID-19 infection, 154 (26.3%) were women. Compared to men, women were significantly older, had a higher prevalence of diabetes and stroke/transient ischemic attack, and were more likely to be on statins on presentation. Men more frequently presented with chest pain, whereas women presented with dyspnea. Women more often had STEMI without an identified culprit lesion than men (33% vs 18%, *P* < .001). The use of percutaneous coronary intervention was significantly higher in men, whereas medical therapy was higher in women. In-hospital mortality was 33% for women and 27% for men (*P* = .22).

**Conclusions:**

In patients presenting with STEMI in the context of COVID-19, the in-hospital mortality rate was 30% and similar for men and women. Lack of an identifiable culprit lesion was common in the setting of COVID-19 for both sexes but more likely in women (1/3 of women vs 1/5 of men). Evaluation of specific underlying etiologies is underway to better define the full impact of COVID-19 on STEMI outcomes and better understand the observed sex differences.

## Highlights

• Patients presenting with STEMI in the setting of COVID-19 had a poor prognosis.
• In-hospital mortality rate was 30%, affecting men and women similarly.
• Lack of an identifiable culprit lesion was common in COVID-19 STEMI for both sexes.
• Women more often had STEMI without an identified culprit lesion compared with men.

## Central Illustration

**Figure:**
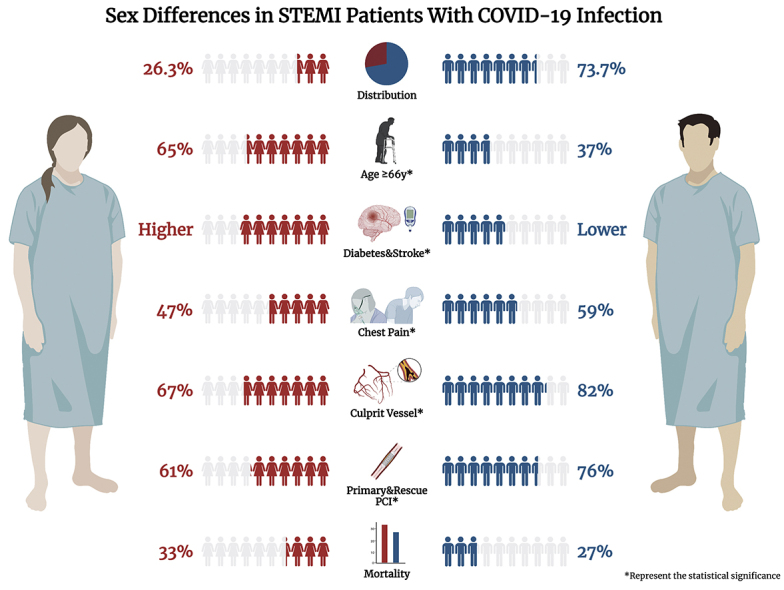

## Introduction

COVID-19 significantly increases the risk of arterial and venous thromboembolic events up to 2-fold, including the risk of myocardial infarction (MI) in the 7 ​days after COVID-19 diagnosis.^1^, ^2^, ^3^ Patients in the early phase of the COVID-19 pandemic who developed ST-segment elevation MI (STEMI) were reported to be at higher risk of mortality (up to 36%),^4^, ^5^, ^6^, ^7^ partly due to the higher prevalence of cardiovascular risks, higher risk of pre-percutaneous coronary intervention (PCI) cardiogenic shock, and lower likelihood of undergoing invasive angiography and mechanical circulatory support in COVID-19 patients with STEMI.^5^^,^^8^

Prior studies in patients with STEMI without COVID-19 infection show sex disparities in STEMI care and outcomes, with some studies reporting a significantly higher risk of mortality in women. The higher mortality in women, particularly young women (<60 ​years of age), was predominantly related to late presentation, delayed diagnosis, and underutilization of evidence-based therapies, including revascularization with primary PCI.^9^, ^10^, ^11^, ^12^, ^13^, ^14^, ^15^ Although men are reported to have more severe disease and higher mortality with COVID-19 infection,^16^^,^^17^ the risk of MI with COVID-19 infection is no different in men compared with women.^18^ Sex differences in patients with COVID-19 infection and MI are limited to only 1 small, single-center study (*n* = 57) in Iran predominantly composed of patients with non ST-elevation MI that reported no sex difference in in-hospital mortality risk.^19^

This prespecified analysis aims to describe sex differences in clinical characteristics, management strategies, and outcomes of STEMI patients with COVID-19 infection using the North American COVID-19 STEMI (NACMI) registry.

## Methods

### Study design

NACMI is a multicenter, prospective, investigator-initiated, observational registry of hospitalized STEMI patients in North America with confirmed or suspected COVID-19 infection, as previously described.^20^ NACMI included a total of 64 sites (12 Canadian and 52 US sites). Institutional review board approval was required at the coordinating center (Minneapolis Heart Institute Foundation) and at each enrolling site. Patients were enrolled from March 1, 2020, to December 31, 2021.

This registry included adult patients (≥18 ​years) with (1) ST-segment elevation in at least 2 contiguous leads (or new-onset left bundle branch block); (2) a clinical correlate of myocardial ischemia; and (3) confirmed or suspected COVID-19 infection. This analysis only included confirmed COVID-19 cases based on positivity by any commercially available test during or 4 ​weeks prior to the index STEMI hospitalization regardless of treatment strategy. Also included patients with in-hospital STEMI presentations with confirmed COVID-19 irrespective of the admission reason. The primary endpoint was in-hospital mortality. Secondary endpoints included stroke, reinfarction, and the composite of mortality, stroke, or reinfarction. Nonfatal events were defined using National Cardiovascular Data Registry (NCDR) CathPCI Registry v4.4 definitions.

### Data collection

Standardized data-collection forms were used, designed using the American College of Cardiology NCDR definitions. Data were collected at the sites and entered into a REDCap database; statistical analysis was performed by the coordinating center (Minneapolis Heart Institute Foundation).

### Statistical analysis

Categorical variables are summarized by count and percentage; continuous variables are summarized by mean ​± ​standard deviation if normally distributed or as median and interquartile range (25th percentile, 75th percentile) if skewed. For categorical data, the Pearson χ^2^ or the Fisher exact test was used; for continuous variables, the *t* test or Wilcoxon rank-sum test was used, as appropriate.

Given a relatively short hospital length of stay, in-hospital mortality is modeled as a binary variable, and relative risk of mortality is estimated from a multivariate robust Poisson regression with a canonical log-link and a robust sandwich estimator of variance to allow for overdispersion in the data. Model covariates included sex, body mass index, abnormal chest x-ray findings, indicator variables for age <66 ​years, White race, current smoker, coronary artery disease, signs of heart failure, diabetes, previous MI, stroke or transient ischemic attack (TIA), PCI or coronary artery bypass grafting, and shock before PCI. The choice of the variables and categories in the model is informed by existing literature, exploratory data analysis, sample size, and the number of adverse events. Model parameters are estimated from imputed data, with missing values approximated by sample medians. Model estimates are reported with their 95% confidence intervals and *P* values.

A *P* value of ​<.05 is considered statistically significant without adjustment for multiplicity. Data were analyzed using R version 4.1.2 (R Foundation for Statistical Computing) in RStudio environment version 2021.09.1 (RStudio, PBC).

## Results

### Clinical characteristics and management strategies

A total of 585 COVID-19 positive patients with STEMI were included in the present analysis, of which 154 (26.3%) were women. Among women, 46% were White, 21% Black, 17% Hispanic, and 9.3% Asian, with similar rates in men. Compared with men, women were older, and more of them had diabetes (53% vs 41%) and stroke/TIA (14% vs 7.4%). There was no other difference in risk factors between sexes including hypertension, dyslipidemia, smoking, body mass index, previous coronary artery disease, and previous MI. Women also had higher statin use prior to presentation (49% vs 32%). Men more frequently presented with chest pain (59% vs 47%), whereas women more frequently presented with dyspnea (56% vs 45%). There was no significant sex-based difference in pre-PCI cardiac arrest, cardiogenic shock, or left ventricular ejection fraction (Table 1).

**Table 1:** Demographics, comorbidities, and clinical features at presentation in STEMI patients with COVID-19 infection by sex.

	Women, *n* ​= ​154	Men, *n* ​= ​431	*P* value
Demographics			
Age, y			<.001
<66	54 (35)	272 (63)	
≥66	100 (65)	158 (37)	
Race			.11
White	69 (46)	217 (52)	
Black	31 (21)	52 (13)	
Asian	14 (9.3)	26 (6.3)	
Hispanic	26 (17)	76 (18)	
Indigenous	2 (1.3)	8 (1.9)	
Other	8 (5.3)	36 (8.7)	
Pre-existing comorbidities			
Hypertension	114 (75)	274 (67)	.058
Dyslipidemia	73 (50)	170 (44)	.17
Diabetes	77 (53)	160 (41)	.01
Smoking history			.36
Current	22 (15)	76 (19)	
Former	36 (25)	108 (28)	
Never	85 (59)	206 (53)	
Body mass index, kg/m^2^	28 ​± ​12	28 ​± ​9	.61
Previous coronary artery disease	39 (27)	100 (26)	.74
Previous myocardial infarction	21 (15)	53 (14)	.76
Previous stroke/TIA	20 (14)	28 (7.4)	.02
Medications on admission			
Aspirin	58 (38)	157 (36)	.79
Statin	75 (49)	136 (32)	<.001
Clinical presentation			
Dyspnea	86 (56)	192 (45)	.02
Chest pain	72 (47)	255 (59)	.008
Syncope	4 (2.6)	18 (4.2)	.38
Signs of heart failure	27 (19)	57 (15)	.25
Cardiomegaly	16 (10)	35 (8.1)	.39
Pleural effusion	15 (9.7)	36 (8.4)	.60
Pulmonary infiltrates	65 (42)	161 (37)	.29
Intubation	44 (30)	97 (24)	.16
Cardiac arrest (pre-PCI)	13 (9.6)	34 (9.0)	.83
Cardiogenic shock (pre-PCI)	23 (17)	52 (14)	.38
In-hospital STEMI	12 (7.8)	27 (6.4)	.56
Left ventricular ejection fraction, %	45 (30, 55)	45 (35, 55)	.90

Values are *n* (%), mean ​± ​standard deviation, or median (interquartile range).

PCI, percutaneous coronary intervention; STEMI, ST-segment elevation myocardial infarction; TIA, transient ischemic attack.

Angiography was not performed in 18% of women and 17% of men. Among those who underwent angiography, women had fewer identifiable culprit lesions than men (67% vs 82%), with one-third presenting with no culprit lesion. Women were more often treated with medical therapy, and men more frequently had primary PCI. In addition, there was no between-group differences in length of stay (Table 2).

**Table 2:** Angiographic features and treatment strategies in STEMI patients with COVID-19 infection by sex.

	Women, *n* ​= ​154	Men, *n* ​= ​431	*P* value
No angiography	28 (18)	72 (17)	.7
Angiographic features			
Culprit vessel	82 (67)	288 (82)	<.001
Door-to-balloon time, min	70 (49, 114)	73 (51, 114)	.6
Treatment^a^			
Primary or rescue PCI	77 (61)	272 (76)	.002
Medical therapy	41 (33)	71 (20)	.003
Thrombolytics	5 (4.0)	11 (3.1)	.6
Coronary artery bypass graft	3 (2.4)	5 (1.4)	.4
Length of stay, d	6 (3, 15)	5 (2, 12)	.10

Values are *n* (%) or median (interquartile range).

PCI, percutaneous coronary intervention; STEMI, ST-segment elevation myocardial infarction.

a For patients undergoing coronary angiography.

### Clinical outcomes

**Figure 1:**
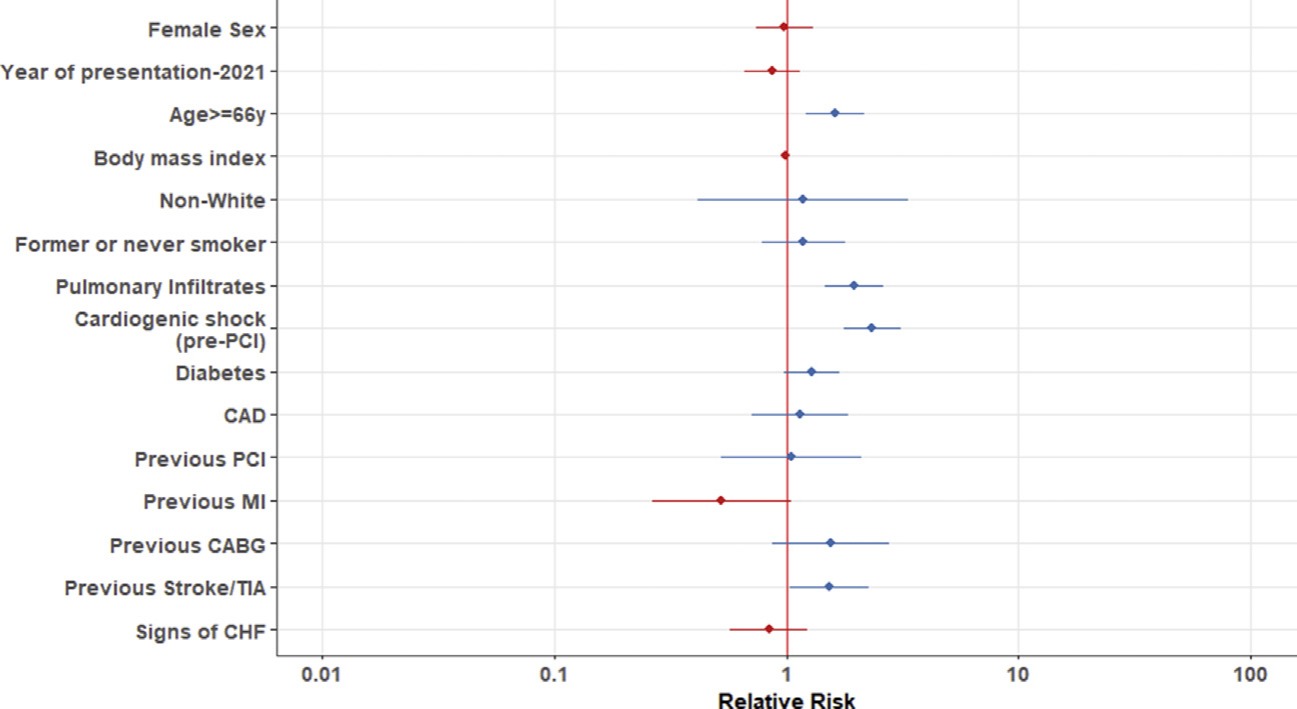
Relative risks for in-hospital mortality in STEMI Patients with COVID-19 infection. CABG, ​coronary artery bypass graft surgery; CAD, coronary artery disease; CHF, ​congestive heart failure; MI, ​myocardial infarction; PCI, ​percutaneous coronary intervention; STEMI, ST-segment elevation myocardial infarction; TIA, transient ischemic attack.

In-hospital mortality was 33% for women and 27% for men. There were no significant sex differences in stroke, reinfarction, or the composite endpoint (Table 3). In the multivariable model, age ≥66 ​years, history of stroke/TIA, pulmonary infiltrates on presentation, and cardiogenic shock (pre-PCI) were independent predictors of in-hospital mortality (Figure 1 and Supplemental Table S1).

**Table 3:** Clinical outcomes in STEMI patients with COVID-19 infection by sex.

	Women, *n* ​= ​154	Men, *n* ​= ​431	*P* value
Mortality, in-hospital	49 (33)	109 (27)	.22
Stroke	2 (1.8)	7 (2.2)	>.999
Reinfarction	4 (3.5)	7 (2.2)	.50
Composite endpoint	54 (40)	116 (34)	.18

Values are *n* (%).

STEMI, ST-segment elevation myocardial infarction.

In-hospital mortality was not significantly different in women and men who (1) did not undergo angiography (60% vs 44%, *P* ​= ​.20); (2) had a culprit lesion (25% vs 18%, *P* ​= ​.19); (3) had no culprit lesion (34% vs 46%, *P* ​= ​.23); (4) were treated medically (29% vs 48%, *P* ​= ​.06); (5) underwent primary/rescue PCI (26% vs 18%, *P* ​= ​.13); or (6) were intubated (61% vs 61%, *P* ​= ​.96).

Among the 165 patients enrolled in 2021 (at which point vaccines were widely available in the United States and Canada), none of the 22 vaccinated patients expired in hospital.

## Discussion

Based on this prospective, multicenter registry, patients presenting with STEMI in the setting of COVID-19 had a poor prognosis, with a 30% overall mortality rate affecting men and women equally. Absence of an identifiable culprit on angiography appears to be more common in the setting of STEMI in COVID-19 but was more likely in women, along with other sex-based differences in risk factors, presentation, and treatment (Central Illustration).

**Central Illustration:**
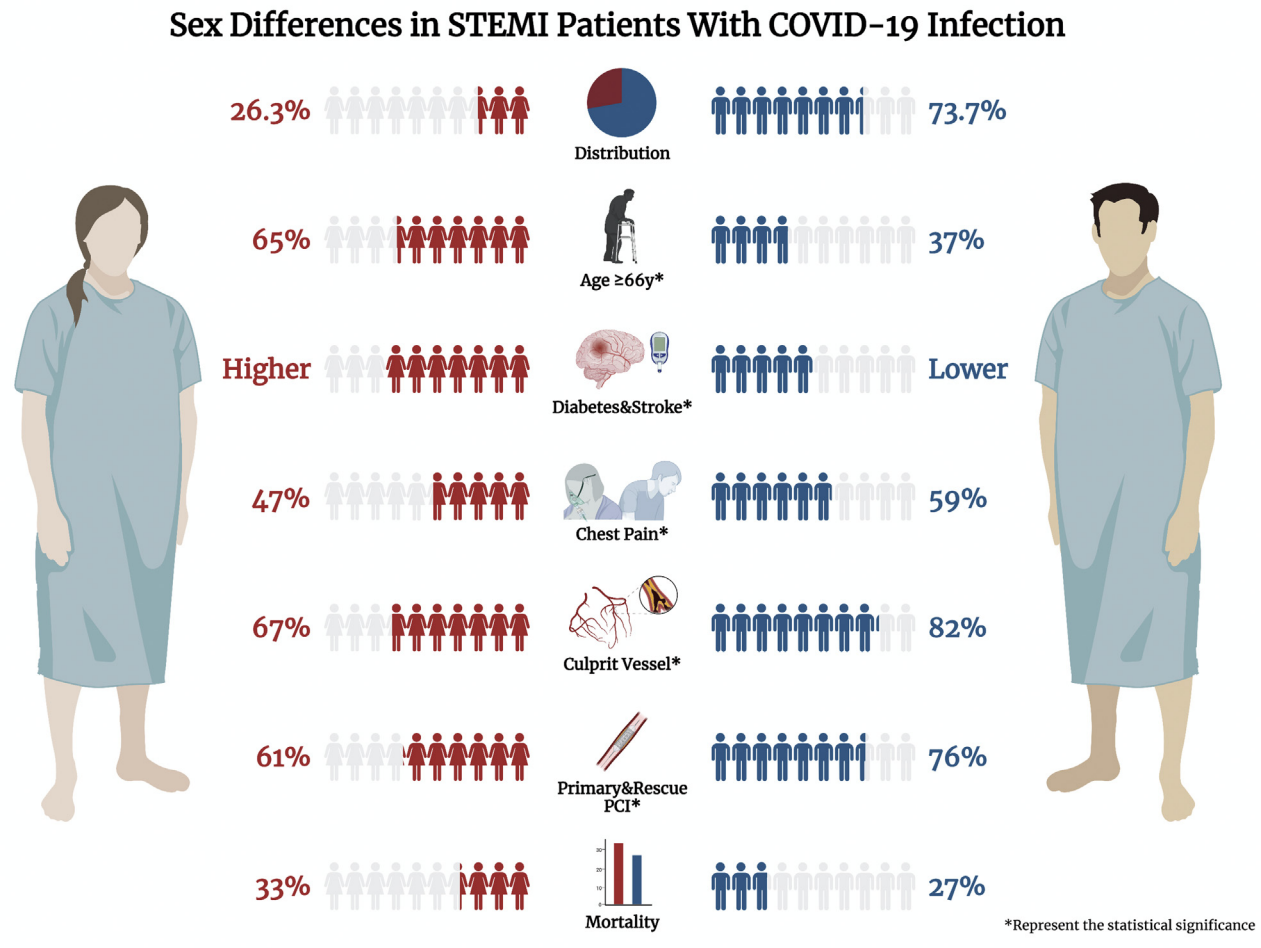
Sex differences in STEMI patients with COVID-19 infection created with BioRender.com.

Prior to COVID-19, mortality rates for STEMI had significantly improved in the past decade, with rates as low as 5%.^4^ This is in stark contrast to the very poor prognosis observed in our registry of patients with STEMI and COVID-19 infection where in-hospital mortality was 33% for women and 27% for men. These findings align with earlier observations that reported in-hospital mortality in STEMI patients with COVID-19 infection ranging from 15% to 36%.^4^, ^5^, ^6^, ^7^ Possible causes of this excess mortality in STEMI associated with COVID-19 include the higher incidence of cardiogenic shock and the presence of pulmonary infiltrates on presentation, suggestive of a more severe COVID-19 infection, which carried a 2-fold higher risk of in-hospital mortality in our study. The combination of the direct effects of the virus, a higher risk of thrombotic lesions and microthrombi, delays in patient presentation, deviations from evidence-based STEMI protocol during the early phase of the pandemic which resulted in treatment delays, and reduced access to angiography likely contributed to the poorer prognosis observed. Further research is needed to understand the impact that COVID-19 vaccinations will have on the incidence and prognosis of STEMI in COVID-19 patients.^21^

No culprit lesion was identified on angiography in 33% of women and 18% of men with STEMI and COVID-19 infection. This is a startling difference to the 3.5%-6.5% rates of MI with nonobstructive coronary artery disease (MINOCA) reported in patients without COVID infection.^22^, ^23^, ^24^, ^25^ Potential mechanisms for STEMI in patients with COVID-19 infection and no culprit lesion on angiography include: microvascular thrombosis/embolization, given that COVID-19 has been described as a state of inflammation and hypercoagulability;^2^ myocarditis, which has been associated with worse prognosis in COVID-19 patients;^26^ stress cardiomyopathy in the setting of severe illness;^27^ demand ischemia in the setting of severe hypoxia;^28^ and electrolyte imbalances common in critically ill patients with COVID-19 infection. Other causes of MINOCA include coronary artery plaque disruption, epicardial coronary spasm, spontaneous coronary artery dissection, and nonischemic cardiomyopathy.^29^ Evaluation of specific underlying etiologies is underway to better define the mechanisms of STEMI in COVID-19 infection. Similarly, MI studies in patients without COVID-19 infection consistently report predominance of MINOCA in women; however, the reason for this remains unknown.^22^, ^23^, ^24^, ^25^ In-hospital mortality was not significantly different in women vs men that did not have angiography performed, had a culprit lesion on angiography, or in those without a culprit lesion on angiography.

Other important sex differences were observed in this study. Women were older and had a significantly higher prevalence of pre-existing diabetes and stroke/TIA. Men presenting with STEMI and COVID-19 more frequently presented with chest pain, whereas women presented more often with dyspnea in the current study,^30^^,^^31^ likely due to the different underlying sex-based mechanisms of STEMI. Similarly, the difference in treatment strategies with more PCI in men and more medical treatment in women likely reflects the higher rates of MINOCA in women compared with men; however, differences in STEMI treatment have been shown to contribute to worse outcomes in women. Therefore, further investigation into the impact of treatment on outcomes is warranted.^11^^,^^13^

## Limitations

The NACMI registry has important limitations common to observational studies when comparing outcomes of subgroups, including measured and unmeasured confounders. Furthermore, clinical events and cause of death were not independently adjudicated, there was no independent angiographic analysis to determine the underlying mechanism of STEMI, and the sample size was limited to detect differences in mortality. Total ischemic time, transfer times for patients presenting to non-PCI hospitals, the reason for not performing angiography, and detailed laboratory data including hypercoagulable biomarkers were not collected. Finally, our study did not capture information regarding COVID-19 variation, and data collection on vaccination status is not complete; therefore, association with outcomes is not possible.

## Conclusions

In the prospective NACMI registry of patients presenting with STEMI and COVID-19, the in-hospital mortality rate was 30% and similar for both men and women. Lack of an identifiable culprit lesion was common in the setting of COVID-19 for both sexes but more likely in women. Evaluation of the underlying mechanisms of STEMI is underway.

## Declaration of competing interest

Financial support was provided by 10.13039/100004374Medtronic Inc, Abbott Cardiovascular Structural Heart Division, 10.13039/501100000106an American College of Cardiology’s Accreditation Services Foundation Committee Grant, and a Saskatchewan Health Research Foundation Grant for the NACMI registry. Dr. Quesada declared receiving grants from NIHK23HL151867. Dr. Garcia declares receiving institutional research grants from 10.13039/100006520Edwards Lifesciences, Boston Scientific, 10.13039/100004374Medtronic, and 10.13039/100011949Abbott Vascular; is a consultant for American College of Cardiology, Medtronic, and Boston Scientific; and is a proctor for Edwards Lifesciences. The remaining authors have nothing to disclose.
