# Supplementary material for: Sex Differences in Clinical Characteristics, Management Strategies, and Outcomes of STEMI With COVID-19: NACMI Registry
Source: J Soc Cardiovasc Angiogr Interv. 2022 May 19;1(4):100360. doi: 10.1016/j.jscai.2022.100360 (PMC9117757; doi:10.1016/j.jscai.2022.100360)
Supplement: Supplemental Table S1 [file mmc1.docx]

**Supplemental Table 1. Estimated Relative Risks for In-Hospital Mortality in STEMI Patients with COVID-19 Infection**

| Variable | Relative Risk | 95% CI Lower | 95% CI Upper | P-Value |
| --- | --- | --- | --- | --- |
| Female sex | 0.98 | 0.72 | 1.32 | .91 |
| Presentation in 2021 | 0.87 | 0.66 | 1.16 | .34 |
| Age ≥66 years | 1.62 | 1.21 | 2.18 | .001 |
| Body mass index | 0.99 | 0.98 | 1.01 | .33 |
| Non-White | 1.18 | 0.45 | 2.52 | .70 |
| Former or never smoker | 1.19 | 0.79 | 1.86 | .43 |
| Pulmonary infiltrates | 1.97 | 1.48 | 2.64 | <.001 |
| Cardiogenic shock (pre-PCI) | 2.35 | 1.69 | 3.22 | <.001 |
| Diabetes | 1.29 | 0.96 | 1.72 | .09 |
| Coronary artery disease | 1.15 | 0.69 | 1.85 | .59 |
| Previous PCI | 1.05 | 0.58 | 1.90 | .87 |
| Previous myocardial infarction | 0.53 | 0.28 | 0.97 | .042 |
| Previous CABG | 1.55 | 0.84 | 2.77 | .15 |
| Previous stroke/TIA | 1.54 | 0.10 | 2.28 | .04 |
| Signs of heart failure | 0.84 | 0.55 | 1.25 | .41 |

CABG = coronary artery bypass graft, PCI = percutaneous coronary intervention, TIA = transient ischemic attack.
